# Coupling Langmuir with Michaelis-Menten—A practical alternative to estimate Se content in rice?

**DOI:** 10.1371/journal.pone.0214219

**Published:** 2019-04-19

**Authors:** Alexandra K. Nothstein, Elisabeth Eiche, Michael Riemann, Peter Nick, Philipp Maier, Arne Tenspolde, Thomas Neumann

**Affiliations:** 1 Karlsruhe Institute of Technology (KIT), Institute for Safety and Environment (SUM), Eggenstein-Leopoldshafen, Baden-Württemberg, Germany; 2 Karlsruhe Institute of Technology (KIT), Institute of Applied Geosciences (AGW), Karlsruhe, Baden-Württemberg, Germany; 3 Karlsruhe Institute of Technology (KIT), Molecular Cell Biology, Botanical Institute Karlsruhe, Karlsruhe, Baden-Württemberg, Germany; 4 Technical University of Berlin (TUB), Institute of Applied Geosciences, Berlin, Germany; Oak Ridge National Laboratory, UNITED STATES

## Abstract

Selenium plays an important, but vastly neglected role in human nutrition with a narrow gap between dietary deficiency and toxicity. For a potential biofortification of food with Se, as well as for toxicity-risk assessment in sites contaminated by Se, modelling of local and global Se cycling is essential. As bioavailability of Se for rice plants depends on the speciation of Se and the resulting interactions with mineral surfaces as well as the interaction with Se uptake mechanisms in plants, resulting plant Se content is complex to model. Unfortunately, simple experimental models to estimate Se uptake into plants from substrates have been lacking. Therefore, a mass balance of Se transfer between lithosphere (represented by kaolinite), hydrosphere (represented by a controlled nutrient solution), and biosphere (represented by rice plants) has been established. In a controlled, closed, lab-scale system, rice plants were grown hydroponically in nutrient solution supplemented with 0–10 000 μg L^-1^ Se of either selenate or selenite. Furthermore, in a series of batch experiments, adsorption and desorption were studied for selenate and selenite in competition with each of the major nutrient oxy-anions, nitrate, sulfate and phosphate. In a third step, the hydroponical plants experiments were coupled with sorption experiments to study synergy effects. These data were used to develop a mass balance fitting model of Se uptake and partitioning. Adsorption was well-described by Langmuir isotherms, despite competing anions, however, a certain percentage of Se always remained bio-unavailable to the plant. Uptake of selenate or selenite by transporters into the rice plant was fitted with the non-time differentiated Michaelis-Menten equation. Subsequent sequestration of Se to the shoot was better described using a substrate-inhibited variation of the Michaelis-Menten equation. These fitted parameters were then integrated into a mass balance model of Se transfer.

## Introduction

It has been known for years that Se is both essential (< 55 μg/d [1]) and toxic (> 400 μg/d [2]) to humans. There is also a growing awareness of Se as a rare and non-renewable resource [3] as well as an environmental pollutant—both geogenically [4], and anthropologically [5]. Currently, Se research faces two equally important, yet entirely diverse goals [3]: (1) securing Se nutrient resources for future generations, and (2), management of Se-enriched waste deposits to protect the environment and improve the quality of life in areas of contamination. For both issues, a quantitative understanding of selenium speciation and abundance on the path from the soil into the plant, and during the partitioning into different plant organs is crucial. This calls for experimental models that integrate a (necessarily reduced) combination of the lithosphere, hydrosphere and biosphere, while, at the same time, remain time defined and controlled with respect to their parameters. While Se transfer has been studied in different models and observational scales, none of these approaches has allowed addressing both the combination of all three spheres while also enabling standardization of parameters:

1. lab-scale modelling, i.e. surface complexation models [6, 7], sequential extraction procedures [8], plant-uptake and incorporation of nutrients which have culminated in the NST model 3.0 [9] has, so far, only addressed one of the spheres;
2. local-scale modelling, i.e. environmental and agronomical case studies, such as Kesterson Reservoir (USA), Punjab (India) [10, 11] or field experiments on phytoremediation [12] and biofortification, [13, 14, 15, 16] have to deal with the parameters present in the respective system and, thus, do not allow for parameter control; Therefore, only partial mass balance models or transfer equations can be derived from such studies.
3. global-scale modelling [5, 17], i.e. oceanic, atmospheric and terrestrial fluxes for global risk prediction, by their very nature, lack the aspect of parameter control as well.

Unfortunately, each of these approaches has its challenges and limitations and it is not possible to combine them into a more comprehensive model as their parameters and approaches vary significantly. For example, there have been many lab-scale studies on sorption behavior of Se onto various soils [8, 18, 19, 20] and minerals, such as [6, 7, 21, 22, 23, 24, 25, 26, 27], as well as plant Se-uptake studies [1, 20, 28, 29] and inner-plant Se transport [30, 31]. While these have greatly increased mechanistic understanding, their focus was not on providing interdisciplinary insight. Although this was attempted with the NST model [9], data for Se have so far not been included and the amount of parameters required, therefore making it an impractical tool for quick estimations of Se content. Moreover, environmental case studies and field experiments both share the drawbacks of having to quantify too many influencing inorganic, organic, anthropogenic and climatic parameters in a non-closed system, such that are specific for a given region. Therefore, the conclusions are not easily transferrable [32]. This lack of data is also the reason why modelling the global Se cycle is still in its early stages and requires more data [5].

So far, little emphasis has been placed on mass balancing the Se transfer and cycling in the Critical Zone [33], which includes soil substrates, soil solutions as well as plants. A suitable example for a soil substrate mineral, is kaolinite—a ubiquitous mineral found in soils of regions with rice agriculture. Kaolinite is considered to be a good model for interactions of anions with clay mineral surfaces, because of the lack of cation exchange interference. While interactions with iron oxides and hydroxides—also a good model for anion exchange and also a frequent mineral found in soils with rice agriculture—have been researched more fully [22, 23, 24, 25, 26], Se adsorption onto clay minerals, particularly onto kaolinite has barely been studied [34], and mechanisms of Se uptake into plants are far from fully understood [35].

In our study, we, therefore, used a simplified experimental model of the Critical Zone to calculate the mass balance in a closed-system approach [36]. First, three compartments (both sources & sinks for Se) were defined: kaolinite was used to represent the lithosphere, a controlled nutrient solution represented the hydrosphere, and rice as the most important staple crop on this planet was used to represent the biosphere. Then, batch experiments were performed to determine adsorption and desorption processes of selenite or selenate onto or from kaolinite in the presence of competing oxyanions typically used for plant fertilization. After that, a combined experimental set-up explored the combination of Se adsorption and simultaneous plant uptake of Se. Finally, all data obtained in this experimental model were used to calculate Se transfer between the three compartments. This allowed the generation of a simplified mass balance model. With this novel approach, we hope to provide a simple and effective tool to estimate the risk of both Se toxicity and deficiency in rice plants at any potentially elevated Se concentrations at local-scale agricultural sites.

## Materials and methods

### Adsorption and desorption behavior of Se on kaolinite

To characterize Se adsorption-desorption behavior, a series of 24-h sorption batch experiments was carried out with 0.5 g kaolinite (AKW KN 83 Amberger Kaolinwerke) in 10 mL of KCl-solution (Merck, p.a. 1.04936.1000) resembling the later used nutrient solution in ionic strength (7.735 mmol L^-1^) and the adsorbent-to-solution ratio. Solutions were spiked with 0–5000 μg L^-1^ Se as either Na_2_SeO_3_ (AlfaAesar 12585) or Na_2_SeO_4_ • 10 H_2_O (VWR BDH Prolabo 302113L). After washing the substrate from the previous adsorption solution with 9.5 mL of double-distilled water, desorption of easily exchangeable Se from the previously adsorbed kaolinite was achieved by using 9.5 mL of 0.1 mol L^-1^ K_2_HPO_4_ (Merck, p.a. 1.05105.1000), a well-known soil extraction step [37]. While the nutrient solution contained a plant-focused anion concentration ratio between nitrate (5000 μM), phosphate (400 μM) and sulfate (750 μM), to study competition effects, the adsorption experiments were repeated with three solutions containing in 0,1 mmol of KCl- (Merck, p.a. 1.04936.1000) and equal concentrations of anions, either an addition of 750 μmol L^-1^ of nitrate as KNO_3_ (Merck p.a. 1.05101.0500), or an addition of 750 μmol L^-1^ sulfate as K_2_SO_4_ (Merck, p.a. 1.05153.0500) or an addition of 750 μmol L^-1^ phosphate as KH_2_PO_4_ (Merck, p.a. 1.04873.1000).

### Combined kaolinite Se-sorption and plant Se-uptake experiments

**Fig 1:**
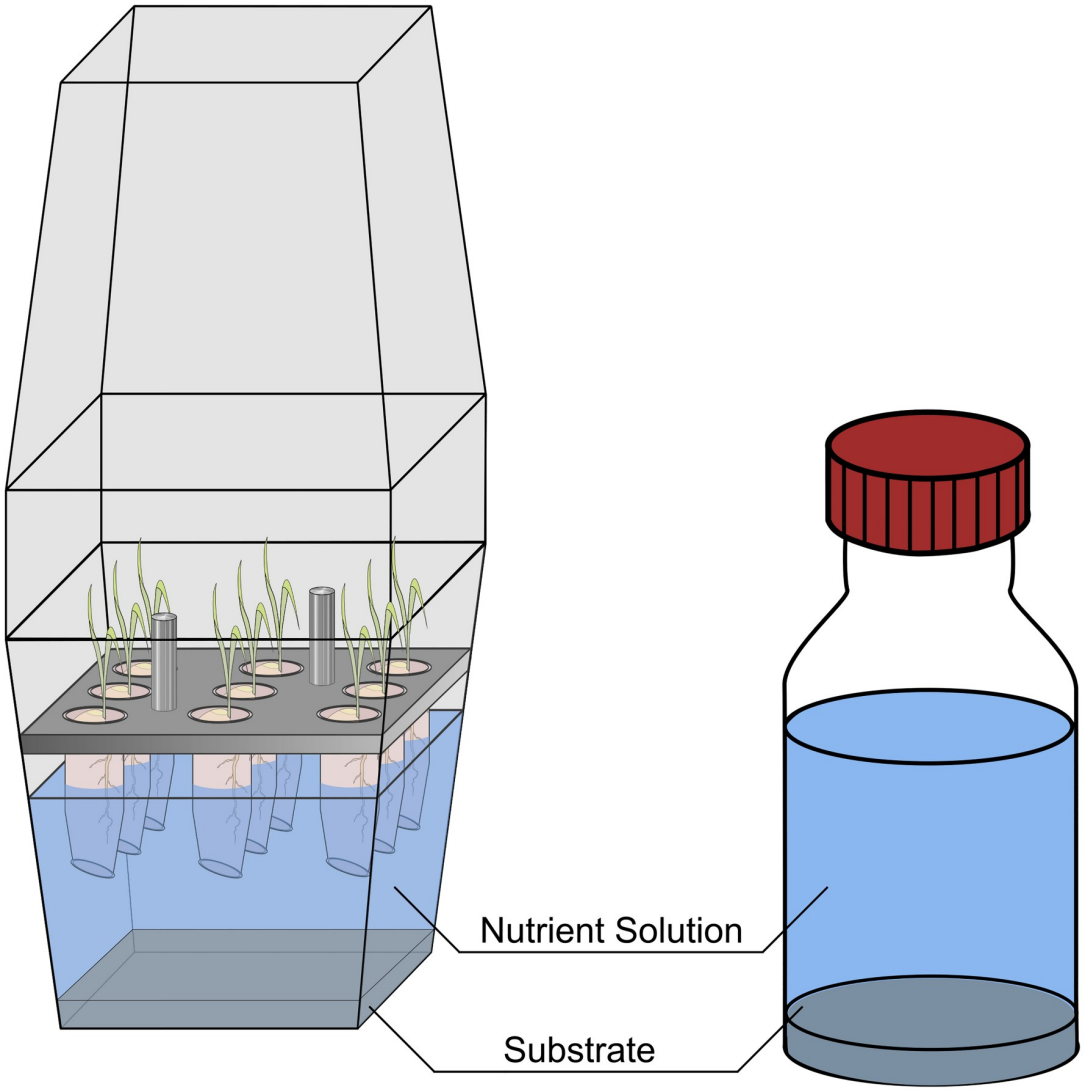
Experimental set-up for the combined kaolinite Se-sorption and plant Se-uptake experiments using 8.5 g kaolinite substrate and 170 mL nutrient solution containing 0–10000 μg L^-1^ Se as selenate or selenite (full experiment in the plant-box on the left and the plant-less control on the right).

To study combined effects of Se sorption processes and Se-uptake by plants, a coupled hydroponic experiment was devised (Fig 1 left). The rice used was *Oryza sativa ssp*. *japonica* (cv. Nihonmasari), cultivated in a direct line obtained in 1991 from NIAR, the National Institute for Agricultural Resources, Tsukuba. Nine caryopses per plant box were dehusked, and surface-sterilized with ethanol (80%) and NaOCl (5%) and germinated in agar-filled (0.7% phytoagar, Duchefa Direct) 1.5-mL reaction-tubes placed in closed Magenta boxes (Sigma Aldrich V8380, V8505 & C0667) and cultivated in the dark at 28°C. After 5 days, their roots reached the nutrient solution (in double-distilled water: 2500 μM Ca(NO_3_)_2_ • 4H_2_O, 375 μM K_2_SO_4_, 325 μM MgSO_4_ • 6H_2_O, 400 μM KH_2_PO_4_, 8 μM H_3_BO_3_, 0.4 μM CuSO_4_, 0.75 μM ZnSO_4_ • H_2_O, 1.2 μM MnSO_4_ • H_2_O, 50 μM CaCl_2_, 0.075 μM Na_2_MoO_4_ • 2H_2_O, 75 μM C_6_H_5_O_7_Fe). The solution, which was spiked with 0–10,000 μg L^-1^ Se as either Na_2_SeO_3_ (AlfaAesar 12585) or Na_2_SeO_4_ • 10 H_2_O (VWR BDH Prolabo 302113L) had by then already pre-equilibrated with the 8.5 g of kaolinite (Amberger Kaolinwerke, AKW KN 83, purity: 88.9%).

To separate plant influence from sorption influence, controls were prepared parallel to the hydroponic experiments in glass bottles containing 170 mL Se spiked nutrient solution and 8.5 g kaolinite, but no plants (Fig 1 right). Boxes and bottles were kept closed for the duration of the experiment in a climate chamber at 70% humidity, with a day-night cycle (daylight: 8 a.m.–4 p.m.) and a transition period of 1 hour for dawn and dusk, respectively, and corresponding temperatures of 28 °C (day) and 22 °C (night).

### Sampling and sample preparation for Se analysis

All plants were harvested (harvest yield: 86% ±6 of all planted caryopses), rinsed externally with Millipore water and separated above the caryopses into root and shoot, which were weighed separately to determine fresh weight and then freeze-dried at 0.05 mbar and -20°C for 24 h to determine plant dry weight. For plant digestion [38], each bulk sample (roots or shoots per plant-box: 0.01–0.1 g) was digested with 1 mL of double-distilled water, 3 mL of concentrated HNO_3_ (subboiled) and 1 mL of 30% H_2_O_2_ (p.a.) Teflon vessels. Each batch of 10 digestion samples included one blank and one plant standard (0.1 g NBS SRM 1567a Wheat Flour) to verify digestion quality. Across all digestions, recovery of this standard was 89% (±7). Each nutrient solution and sorption solution was sampled twice into 20-mL patho-vessels (Böttger 08-313-1001); 15 mL of solution sample were frozen at -20°C until anion analysis with IC, the other 15 mL were acidified with 50 μL of HNO_3_ (VWR 20429.320 p.a. sub-boiled) and stored at 4 °C for ICP-MS measurement of Se.

### Se analysis in digestion samples with HG-FIAS

Total Se-content of the roots and shoots of plants harvested from all three set-ups was analysed with HG-FIAS (Hydride Generation Flow Injection Atomic Absorption Spectroscopy; Perkin Elmer AAnalyst200, FIMS-400 Hydride Generation System). Total Se in samples was completely reduced to selenite for 15 min in 6 M Hg-free HCl (Merck, 37%, 1.13386.2500) in a water bath pre-heated to 75 °C and then diluted to 1 M HCl with double-distilled water and measured with HG-FIAS. For calibration, 10 mL of Se standard solution (1000 μg L^-1^ Se, Roth Rotistar ICP) were reduced to selenite in 6 M HCl in the same way. From this solution, calibration concentrations of 0.5, 0.75, 1, 2, 4, 5, and 6 μg L^-1^ Se were prepared with 1 M Hg-free HCl. Reduction quality and drift correction was assessed using a multi-element drinking water standard (PromoChem Trace Metals QCP 050–1 and QCP 050–2 combined, with 252 μg L^-1^ Se). This drinking water standard recovery was 108% (±7) across all measurements.

### Anion analysis of phosphate, nitrate and sulfate with IC

Ion chromatography (IC) analysis of anions was conducted using a Dionex ICS 1000 with an IonPac AS14 column coupled with an IonPac AG14 pre-column and an eluent of 3.5 mmol L^-1^ Na_2_CO_3_ and 1.0 mmol L^-1^ NaHCO_3_ (flow rate: 1.1 mL/min). After thawing, samples were diluted by factor 4 and measured after calibrating with a multi-ion IC-standard calibration solution (Alfa Aesar, Specpure) diluted to 2, 5, 10, 20, 40 mg L^-1^ Cl^-^ and 4, 10, 20, 40, 80 mg L^-1^ NO_3_^-^ and SO_4_^2-^ and 6, 15, 30, 60, 120 mg L^-1^ PO_4_^3-^. Using an Anion Self-Regenerating Suppressor (ASRS 300), conductivity of samples was detected at an applied current of 25 mA for an injection volume of 25 μL. Linear drift correction was applied using re-measurements of calibration standards after every 10 samples. Overall analysis quality was confirmed using a river water standard (BATTLE-02, Environment Canada; 42.4 mg L^-1^ Cl^-^, 0.194 mg L^-1^ F^-^, 149 mg L^-1^ SO_4_^2-^) measured with a dilution factor of two. This river water standard retrieval was 105% (±2) across all measurements.

### Analysis with ICP-MS

Analysis of ^77^Se, ^78^Se, and ^82^Se was performed using an inductively coupled plasma mass spectrometer (ICP-MS) X-Series 2 (Thermo Fisher Scientific) in CCT-Ed mode (Collision Cell Technology—Energy Discrimination). Five mL of sample were diluted by factors of 2–10 in 1% subboiled HNO_3_. Each sample was spiked with 50 μL of internal standard (10 μg L^-1^ Sc, Merck 1.70349.100; 10 μg L^-1^ Rh, Merck 1.70345.0100; 10 μg L^-1^ In, Merck 1.70324.0100; 10 μg L^-1^ Tm, Merck 1.70361.0100) for internal drift correction. Calibration was carried out using an ICP-Se standard solution (Merck 1.70350.0100) in concentrations between 0.5 and 1000 μg L^-1^ Se. Samples were measured with a dilution factor of 2–5. Linear drift correction was applied using re-measurements of calibration standards after every 10 samples and overall analysis quality was confirmed using a trace metal standard CRM-TMDW-A (High Purity Standards) with a dilution factor of five. This trace metal standard retrieval was 107% (±6) across all measurements of all Se isotopes.

### Modelling Se adsorption onto kaolinite

Using Origin Pro 2015G, the experimental Se adsorption data was fitted to a Langmuir isotherm model (Eq 1) in which q describes the adsorption density of the solute in g/kg, q_max_ describes the maximum surface density of the solute in g/kg, K_L_ is the conditional Langmuir equilibrium constant and c describes the concentration of solute in solution in g L^-1^ [39].

$$q=q_{max}\frac{K_{L}∙c}{1+K_{L}∙c}$$ (1)

When subtracting the amount of the Se exchangeable by K_2_HPO_4_, the amount of irreversibly adsorbed Se was obtained, which was also modelled using Eq 1.

#### Modelling Se uptake into rice plants

Plant-Se content appears to be the result of transporter protein activity of the sulfate and phosphate transporters [35]. With the experiment duration of 19 days suggesting steady-state kinetics, plant Se content was modelled using a Michaelis-Menten model (Eq 2), with v describing the initial reaction velocity, v_max_ determining the maximum reaction velocity, c_s_ describing the substrate concentration in solution and k_m_ as the Michaelis-Menten constant for the binding of the substrate to the enzyme [40].

$$v=\frac{v_{max}∙c_{s}}{K_{m}+c_{s}}$$ (2)

As this study’s focus is not the parameters of enzyme kinetics, but the resulting plant-Se content c_p_ and the maximum plant-Se content c_max_, the time component t in the velocity fraction is reduced for v (Eq 3) and v_max_ (Eq 4), similar to the original NST model [41].

$$v=\frac{c_{p}}{t}$$ (3)

$$v_{max}=\frac{c_{pmax}}{t}$$ (4)

This provides the non-time differentiated Michaelis-Menten model used to fit the experimental data of Se content in rice seedlings used in this study (Eq 5).

$$c_{p}=\frac{c_{pmax}∙c_{s}}{K_{m}+c_{s}}$$ (5)

Furthermore, the substrate excess inhibition version of the Michaelis-Menten equation (Eq 6), reduced as shown in Eqs 3 and 4, was tested on the Se sequestration within the plant, with the parameter k_i_ describing the dissociation of the substrate from the transporting enzyme.

$$c_{p}=\frac{c_{pmax}∙c_{s}}{K_{m}+c_{s}∙(1+\frac{c_{s}}{k_{i}})}$$ (6)

To increase model reliability, data from Se uptake into rice seedlings under similar conditions previously published [42] were also included in the model.

## Results

### Se adsorption onto kaolinite—Influences of nutrient solution anions

**Fig 2:**
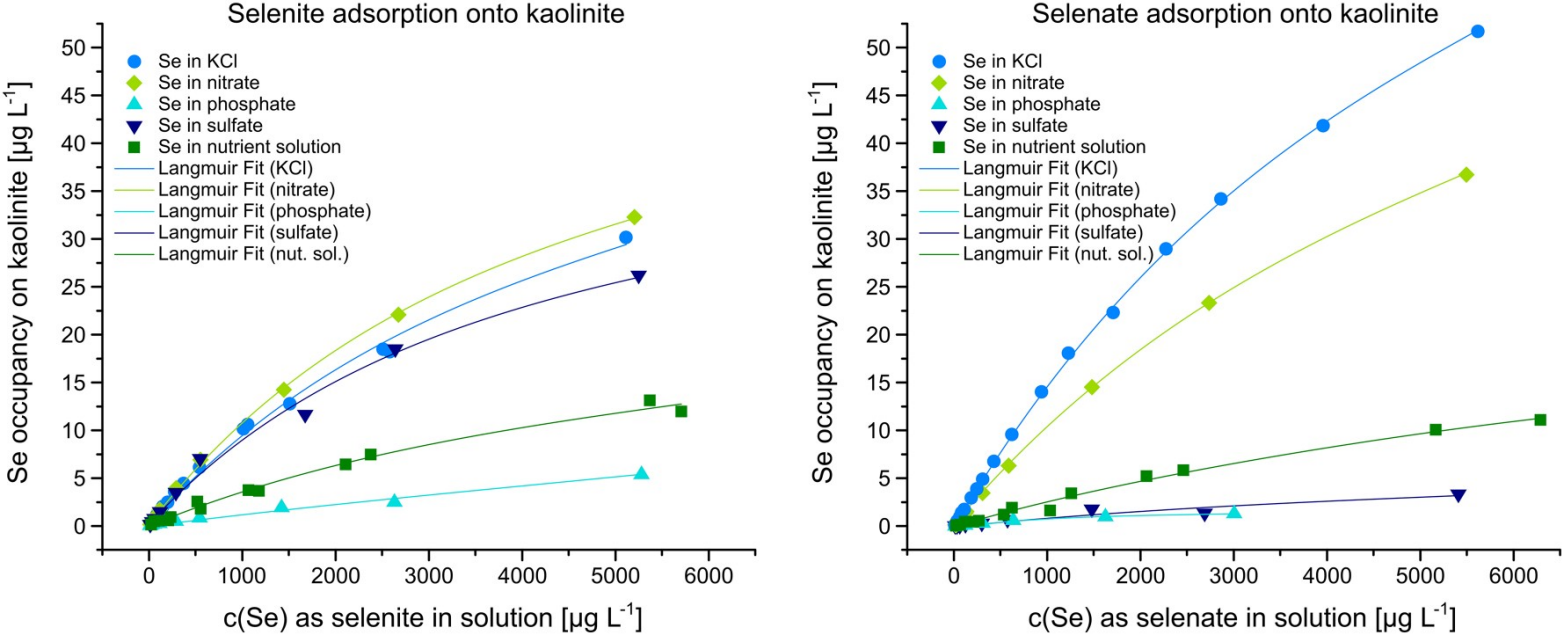
Adsorption of Se onto kaolinite modelled with the Langmuir equation (Eq 1). Values for fitting parameters q_max_ and K_L_ given in Table 1.

Compared to the adsorption of pure selenite onto kaolinite in the presence of 7735 μM KCl, neither the presence of 750 μM N as nitrate in KCl solution nor the presence of 750 μM S as sulfate in KCl solution had any significant influence (±14%) on selenite adsorption (Fig 2, left). The presence of 750 μM of P as phosphate in KCl solution, on the other hand, lowered the selenite adsorption down to 12–38% (x_mean_ = 20% ±9) compared to pure selenite adsorption in KCl. In the presence of nutrient solution, selenite adsorption was 36% ±10 of the pure selenite adsorption in KCl. As the nutrient solution contained 400 μM P as phosphate, the resulting inhibition of selenite adsorption seen in the presence of nutrient solution could theoretically be calculated to be 70% phosphate-induced (Fig 2, left).

Compared to the adsorption of pure selenite onto kaolinite in the presence of 7735 μM KCl, selenate adsorption, however, was inhibited by each of the ions to various degrees (Fig 2, right). While selenate adsorption was moderately lowered down to 66–80% (x_mean_ = 72% ±5) by 750 μM N as nitrate in KCl solution, it was very strongly reduced down to 2–10% (x_mean_ = 5% ±3) and 2–8% (x_mean_ = 6% ±2) by the equivalent amounts of S as sulfate in KCl solution and P as phosphate in KCl solution, respectively. However, in the presence of nutrient solution, selenate adsorption was 15% ±5 of the pure selenate adsorption in KCl and, therefore, inhibited less in the combination of 5000 μM of N as nitrate, 400 μM P as phosphate and 750 μM as sulfate than with 750 μM of each ion individually (Fig 2, right).

**Fig 3:**
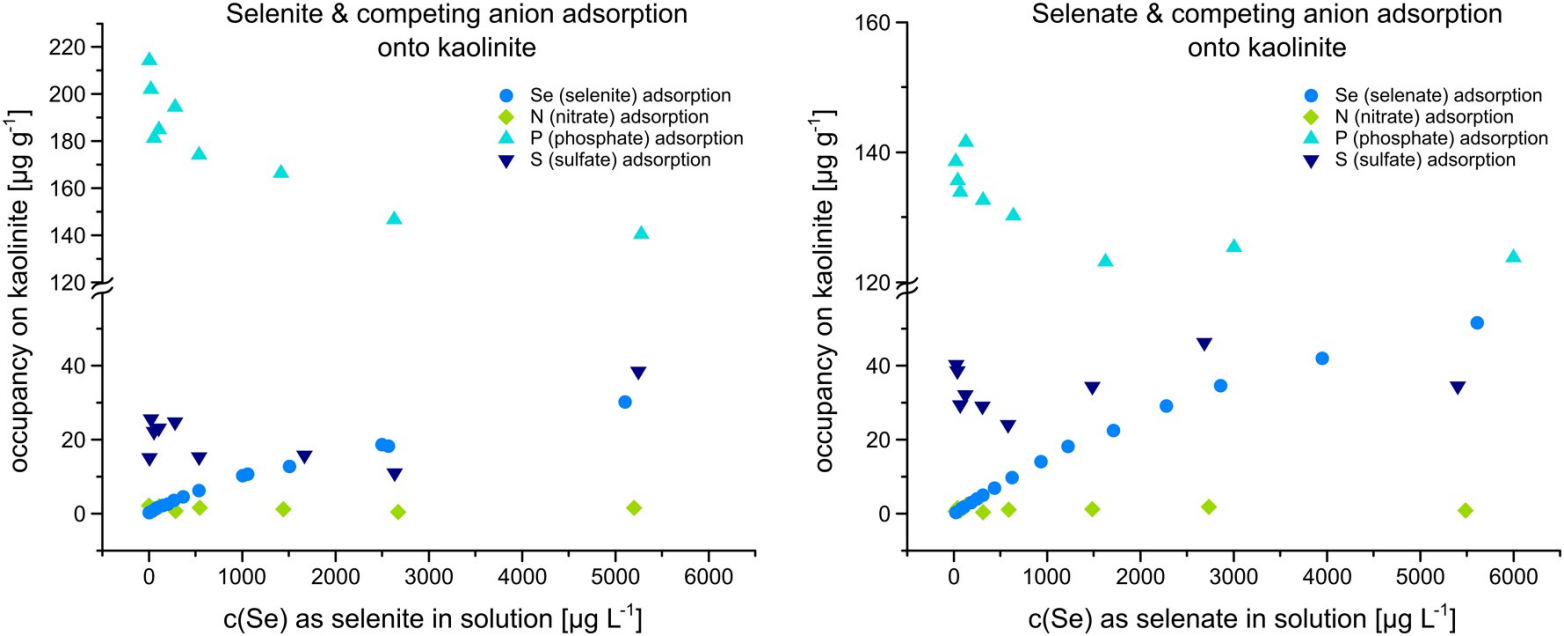
Adsorption of Se and competing anions nitrate, phosphate and sulfate onto kaolinite.

When calculating the adsorption of the competing ions onto the kaolinite surface (Fig 3), nitrate adsorption was 1.4 mg kg^-1^ ±0.6 and 1.1 mg kg^-1^ ±0.6 in the presence of selenite and selenate, respectively, and can, therefore, be regarded as remaining constant regardless of initial Se concentration. Sulfate adsorption was 21.2 mg kg^-1^ ±8.2 and 34.1 mg kg^-1^ ±6.7 in the presence of selenite and selenate, respectively, and can, therefore, also be considered constant regardless of initial Se concentration. Phosphate (Fig 3, left) adsorption however, showed a decrease in adsorption onto kaolinite of 178 mg kg^-1^ ±24 with a 34-% decrease from 214 to 141 mg kg^-1^ adsorption with the increase of initial Se as selenite. This was not observed with statistical significance in the presence of Se as selenate (Fig 3, right), where phosphate adsorption remained constant at 132 mg kg^-1^ ±7.

**Fig 4:**
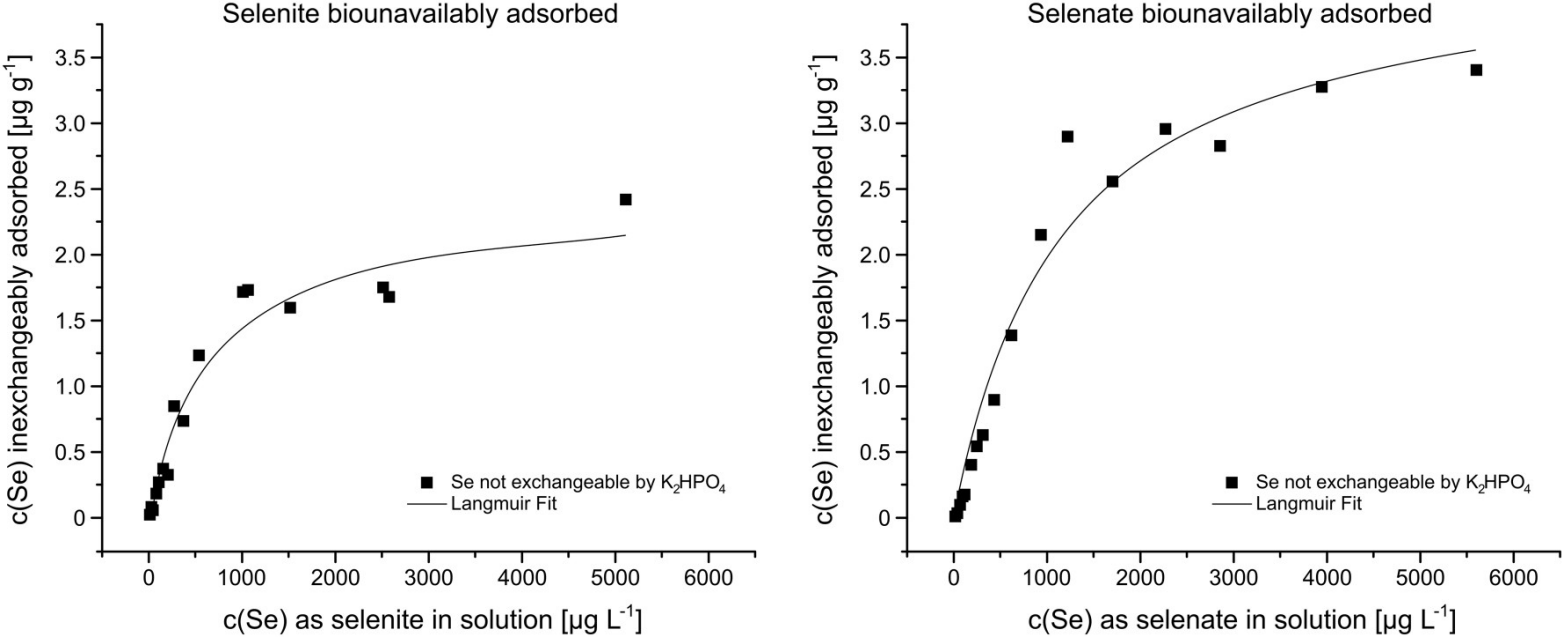
Selenite and selenate adsorption which was inexchangeable by K_2_HPO_4_ was considered biounavailable.

In the subsequent de-sorption experiment, previously adsorbed Se was shown to be exchangeable using K_2_HPO_4_; 85.1% ±5.4 and 88.5% ±3.5 were exchangeable by K_2_HPO_4_ as selenite and selenate, respectively, with no trend regarding the initially applied Se concentration. The remaining inexchangeably adsorbed Se (S1 Table) was also fitted with the Langmuir isotherm (Fig 4) with good correlations (Table 1) of R^2^ = 0.93 and R^2^ = 0.96 for selenite and selenate, respectively.

**Table 1:** Values and statistics for the experimental data fitting of selenite and selenate adsorption onto kaolinite.

Se adsorption solution mode	q_max_		K_L_		fitting statistics	
	value	SD	value	SD	χ^2^ red.	corr. R^2^
**selenite**						
pure Se adsoption	60.29	4.32	1.86 E-4	2.04 E-5	0.3832	0.9948
inexchangeable Se	2.42	0.20	1.50 E-3	3.57 E-4	0.0396	0.9336
nitrate & Se	60.64	1.63	2.17 E-4	1.01 E-5	0.0586	0.9996
phosphate & Se	33.22	4.89	3.63 E-5	3.15 E-5	0.0499	0.9841
sulfate & Se	46.99	5.79	2.37 E-4	5.23 E-5	0.9096	0.9895
nutrient solution & Se	28.53	2.98	1.41 E-4	2.31 E-5	0.1538	0.9914
**selenate**						
pure Se adsoption	112.83	2.12	1.51 E-4	4.26 E-6	0.0733	0.9997
inexchangeable Se	4.29	0.31	8.53 E-4	1.61 E-4	0.0618	0.9636
nitrate & Se	85.24	0.37	1.38 E-4	9.27 E-7	0.0011	0.9999
phosphate & Se	2.05	0.12	5.77 E-4	6.87 E-5	0.0009	0.9965
sulfate & Se	7.99	4.83	1.19 E-4	1.06 E-4	0.1107	0.9111
nutrient solution & Se	32.09	4.99	8.64 E-5	1.86 E-5	0.1126	0.9913

### Se uptake into the seedling—Modelling total uptake vs. Se partitioning

**Fig 5:**
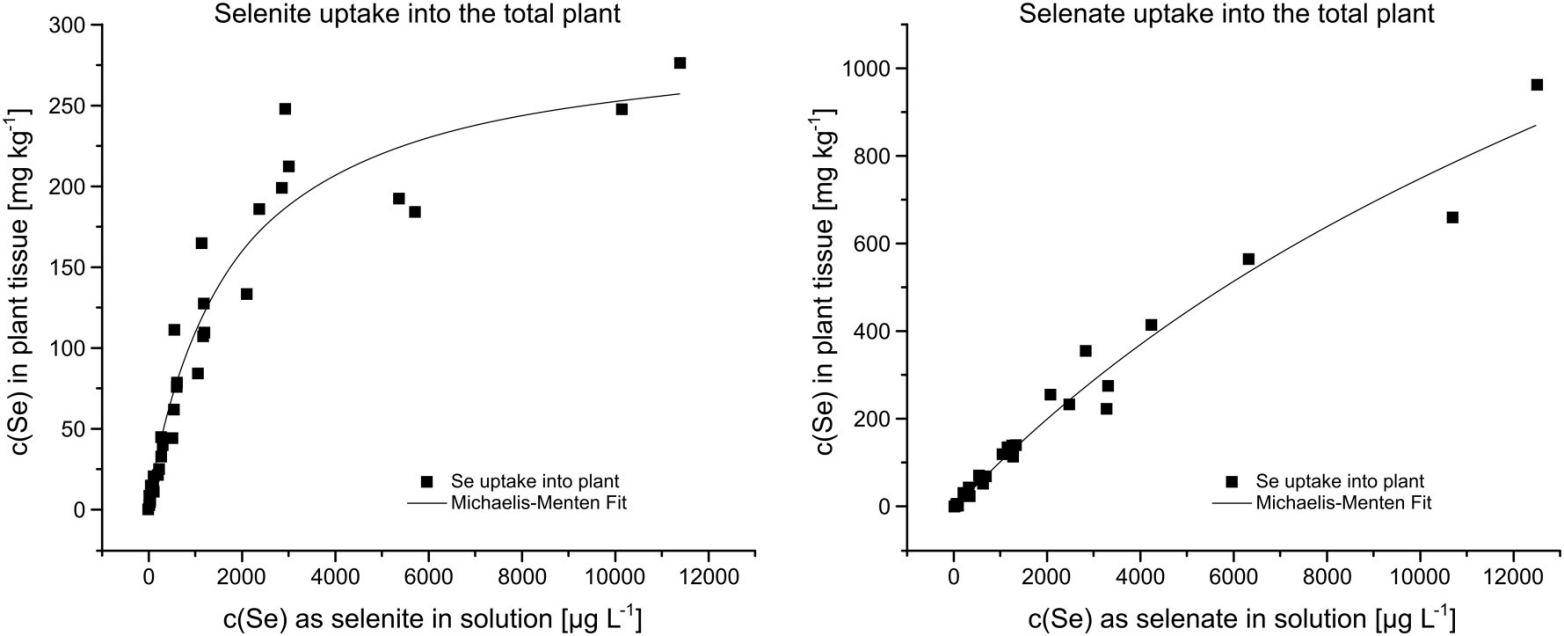
Uptake of Se into the total rice plant seedling modelled with the non-time differentiated Michaelis-Menten equation (Eq 5).

When accounting purely for the Se amount taken up by the plant over the course of 14 days of Se contact, regardless of its partitioning inside the plant tissue (Fig 5), plant uptake increased with the addition of solution-Se content for both selenite and selenate. When Se was added as selenite, uptake into the plant increased more strongly between the addition of 0–2400 μg L^-1^ Se, while it increased with lesser inclination between additions of 2400–12 000 μg L^-1^ Se. Supplying the solution with 2377 μg L^-1^ Se as selenite, for example, resulted in 186 mg kg^-1^ ±13 total plant Se concentration, while supplying the solution with 11 378 μg L^-1^ Se—a concentration more than 4 times higher—led to a total plant Se content of 276 mg/kg ±14, which is an increase of only 1.5. For the addition of Se as selenate, however, plant uptake increased steadily with Se solution concentration between 0 and 12 000 μg L^-1^ Se. Supplying the solution with 12 430 μg L^-1^ Se resulted in total Se plant content of 962 mg kg^-1^ ±32.

These data were fitted using the non-time differentiated Michaelis-Menten equation (Eq 5). Fitting selenite uptake (Fig 5, left) produced c_pmax_ = 296 mg kg^-1^ ±16 and K_m_ = 1708 ± 230 with a good correlation (R^2^ = 0.94). Fitting selenate uptake (Fig 5, right) produced c_pmax_ = 2428 mg kg^-1^ ±378 and K_m_ = 22240 ±4661 and showed a better correlation (R^2^ = 0.97).

**Fig 6:**
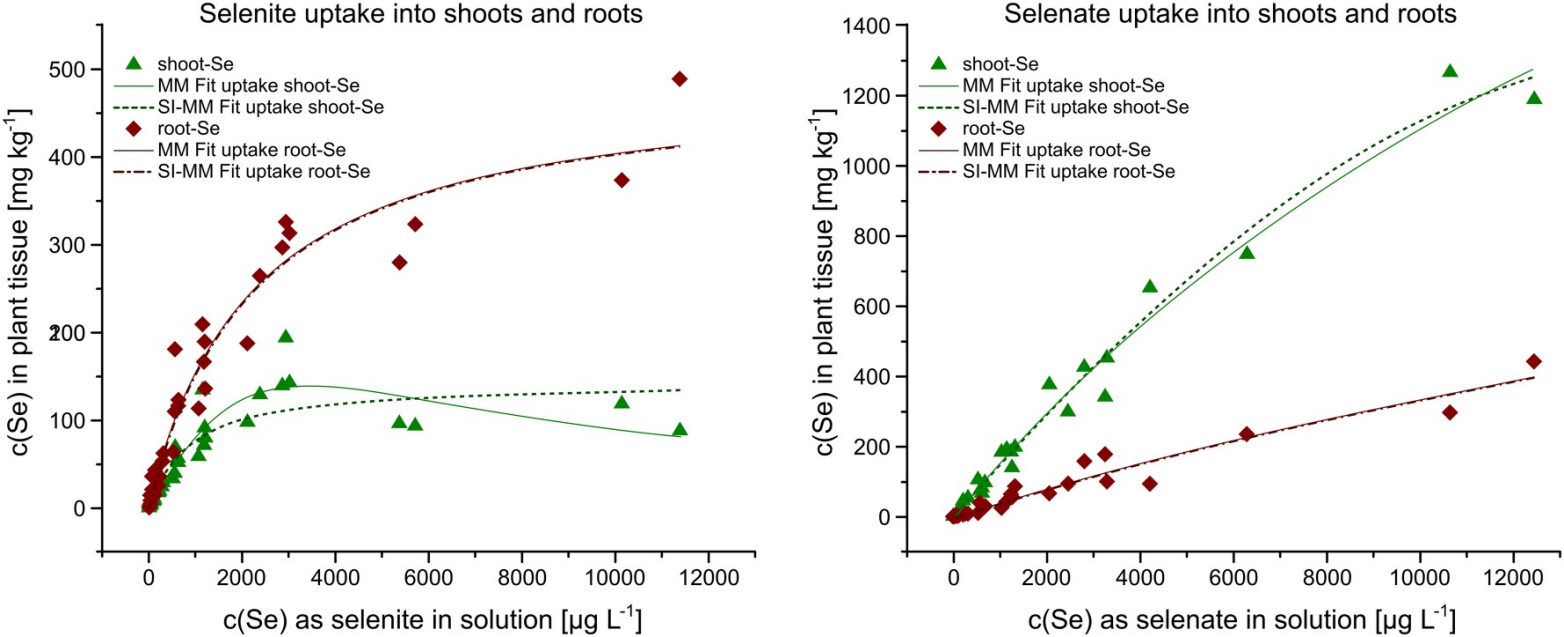
Uptake of Se into shoots and roots of rice plant seedlings modelled with the non-time differentiated Michaelis-Menten (MM) equation (Eq 5) and its substrate-inhibited (SI-MM) variation (Eq 6). SI-MM fitting for the roots was nearly identical to the MM fit for both selenite and selenite.

However, differentiating Se uptake into shoots or roots enabled more detailed fittings. When comparing fittings of the non-time differentiated Michaelis-Menten equation with the fittings of its substrate-inhibited variation (Fig 6), both allowed for good fitting results with corr. R^2^ > 0.8 (Table 2). For the roots, irrespective of the Se speciation, the Michaelis-Menten fitting and its substrate-inhibited variation converged into nearly identical fits (Fig 6, Table 2). Selenium uptake into the shoots, however, yielded a better fit for the substrate-inhibited Michaelis-Menten equation (Table 2)—particularly if Se was applied as selenite (corr. R^2^ = 0.90 vs. 0.83).

**Table 2:** Values and statistics for the experimental data fitting of selenite and selenate uptake into the total plant, shoots and roots using the non-time differentiated Michaelis-Menten (MM) equation (Eq 5) and its substrate-inhibited (SI-MM) variation (Eq 6).

plant tissue model	c_pmax_		K_M_		K_i_		fitting statistics	
	value	SD	value	SD	value	SD	χ^2^ red.	corr. R^2^
**selenite**								
plant-MM	295.5	16.1	1707.9	230.1	-	-	350.30	0.9481
shoot-MM	143.6	12.4	856.2	216.3	-	-	433.1	0.8320
shoot-SI-MM	1113.6	1565.7	12014.8	18451.9	975.6	1571.4	259.73	0.8993
root-MM	492.5	28.3	2205.1	295.1	-	-	788.3	0.9499
root-SI-MM	492.5	28.6	2205.1	298.6	5.2 E108	0	807.1	0.9487
**selenate**								
plant-MM	2428.3	377.7	22239.9	4661.1	-	-	1070.9	0.9731
shoot-MM	3608.3	441.4	22770.2	3735.9	-	-	1359.1	0.9840
shoot-SI-MM	1.1 E6	2.4 E8	7.7 E6	1.7 E9	47.7	1034.1	1282.2	0.9849
root-MM	1934.2	847.6	47882.1	24774.6	-	-	410.0	0.9476
root-SI-MM	1934.2	857.7	47882.0	25067.7	9.0 E93	0	419.8	0.9436

## Discussion

### The mass balance fitting model

To calculate the mass transfer concentrations from the experimental closed-system model for a simple mass balance fitting model, the separately studied processes of adsorption, bioavailable exchange and plant uptake were combined. This approach was considered appropriate, as in practical application, the process of plant growth is slower than adsorption or desorption processes [43] and the growing rice plant, therefore, is placed in an already equilibrated system similar to the experimental model [42]. The appropriateness of the fitting equations can, therefore, be evaluated separately for each process.

### Se adsorption and desorption

The fact that Se ad- and desorption can be fitted with Langmuir very well—independently of the competing ions—is fortunate for a simple model, as attempted with this study and also shown in other studies [43, 44]. However, as previously noted [45], Langmuir fitting itself allows for no information on mechanistic properties during the sorption process. What this study was able to show, however, was that there is a notable amount of inexchangeably bound selenite and selenate (15 and 10%, respectively), which cannot be exchanged—even by phosphate anions. This allows the conclusion that similar to iron-oxide surfaces [24], clay mineral surfaces allow for innersphere-complexation for Se-anions, with selenite bound as a binuclear bidentate complex and selenate bound as a mononuclear monodentate complex as has previously been suggested [7, 25]. This explains the differences between selenite and selenate found when comparing adsorption behavior in competition with other nutrient anions. While the binuclear bidentate selenite—kaolinite complex formation is only notably hampered by the presence of phosphate, the mononuclear, monodentate selenate—kaolinite complex formation is influenced by all nutrient anions, particularly sulfate and phosphate. Nitrate is believed to act as an inhibitor mainly because of its size and high. In all cases, however, a large portion of Se can be exchanged again from the mineral surface and is, therefore, considered 85–89% bioavailable and highly mobile for kaolinite surfaces. In contrast, soils with a high content of iron oxides or hydroxide minerals show such a high affinity for Se anions that environmental Se concentrations can be considered irreversibly bound [22, 23, 24, 25, 26, 27, 42].

### Se uptake and distribution within the plant

As discussed previously in the literature [35], selenite and selenate differ not only in the transporters with which they are taken up into the plant, but also the transportation pathways within the plant. This is why selenate is preferentially partitioned to shoots, while selenite uptake is preferentially partitioned to roots [42], which is also apparent in Fig 6. With the main reason for modelling the Se cycle being to understand the uptake of Se into animals and humans [5], modelling the differentiation between the plant compartments is necessary.

Similar to the all-encompassing Langmuir isotherm equation, applying the Michaelis-Menten equation not to a specific enzyme, but to the entire process of anion uptake into the plant, cannot provide detailed mechanistic understanding. However, even with this in mind, the data show that there is a systematic difference between shoots and roots of the rice plant that affects both selenite and selenate, because the Michaelis-Menten equation (Eq 5) best describes resulting root-Se, while the substrate-inhibited equation (Eq 6) best describes resulting shoot-Se. Although this applies regardless of the Se speciation (Fig 6), selenite transportation into the shoot shows a stronger tendency toward the substrate-inhibited fitting than selenate transportation (Table 2). This allows the conclusion that some form of substrate-excess inhibition occurs not during the uptake of Se, but at some compartmental boundary within the plant between root and shoot, most likely at the boundary to the xylem.

### Applicability of the model

The main advantage of this experimental approach and the resulting mass balance fitting model lies in its simplicity. When confronted with the task of estimating Se content in rice plants for the purpose of toxicity estimation, this model only requires the knowledge of either Se content in the soil or the soil solution, which can easily be obtained. Moreover, a large, previously unexplored range of Se concentrations was covered in this study and the Se distribution between roots and shoots was easily calculable.

Unfortunately, the data are not extensive enough to allow for a fully calculated approach on the effects of competing anions in varying concentrations, as this would have required many iterations of competing sorption experiments. However, this is also not all that useful in practical application, as plants generally require all nutrient anions in abundance to be available for optimal growth. Influences of varying ion concentrations when applying fertilization were not the core subject of study, as these are at least partially covered in other studies [9, 28, 29].

While this mass balance fitting model presents a simplistic approach to modelling Se in different compartments of the Critical Zone, this model can easily be expanded in future studies to cover i.e. other minerals and grown, grain-bearing rice plants. Similar approaches can be applied to other plant species as well. Applicability for this model can be extended beyond the quick estimation of Se content in a specific agricultural setting. Using this approach, a global map of Se content in soil can be overlaid with the expected concentrations of Se in plants, as the bioavailability is included in the model approach. A small database for a number of soils and speciation-dependent uptake models for other ions, such as arsenic, cadmium etc. could provide the basis for this global modelling.

## Supporting information

S1 Table Experimental data of selenite and selenate sorption onto kaolinite in the presence of 0.1 M KCl and subsequent desorption of selenite and selenite from kaolinite using K_2_HPO_4_. (PDF)

S2 Table Experimental data of selenite sorption onto kaolinite in the presence of 0.1 M KCl and 750 μM nitrate, phosphate or sulfate. (PDF)

S3 Table Experimental data of selenate sorption onto kaolinite in the presence of 0.1 M KCl and 750 μM nitrate, phosphate or sulfate. (PDF)

S4 Table Experimental data of selenite and selenate sorption onto kaolinite in the presence of nutrient solution. (PDF)

S5 Table Experimental data of selenite uptake into rice seedlings in the presence of nutrient solution and kaolinite. (PDF)

S6 Table Experimental data of selenate uptake into rice seedlings in the presence of nutrient solution and kaolinite. (PDF)
