# Supplementary material for: Coupling Langmuir with Michaelis-Menten—A practical alternative to estimate Se content in rice?
Source: PLoS One. 2019 Apr 19;14(4):e0214219. doi: 10.1371/journal.pone.0214219 (PMC6474650; doi:10.1371/journal.pone.0214219)
Supplement: S1 Table — (PDF) [file pone.0214219.s001.pdf]

S1 Table: Experimental data of selenite and selenate sorption onto kaolinite in the presence of 0.1 M KCl and subsequent desorption of selenite and selenite from kaolinite using K_2HPO_4

pure selenite adsorption

	c(Se)_init [µg/L]	SD [µg/L]	pH_init [-]	sol-Vol [mL]	m_kaolinite [g]	c(Se)_end [µg/L]	SD [µg/L]	pH_end [-]	c(Se)_loss [%]	c(Se)_sorp [µg/g]	Vol_sol [mL]	pH_desorp [-]	c(Se)_desorp [µg/L]	SD [µg/L]	c(Se)_fixed [µg/g]	desorp/sorp [%]
	10,89	0,05	5,19	0,01	0,5004	4,21	0,01	6,59	61,36	0,15	9,50	8,01	5,47	0,03	0,03	22,21
	26,50	0,06	5,25	0,01	0,5008	10,04	0,02	6,63	62,10	0,37	9,50	7,93	13,14	0,08	0,09	24,15
	47,19	0,64	5,30	0,01	0,5001	19,39	0,04	6,67	58,92	0,63	9,50	8,03	26,17	0,16	0,07	10,59
	81,25	0,29	5,12	0,01	0,5004	30,89	0,12	6,67	61,98	1,13	9,50	7,96	44,03	0,77	0,19	16,95
	106,75	1,99	5,34	0,01	0,5000	41,99	0,30	6,65	60,66	1,46	9,50	7,99	55,00	0,18	0,28	19,30
	155,23	1,21	5,22	0,01	0,5005	64,95	0,57	6,70	58,16	2,03	9,50	7,99	77,21	0,24	0,38	18,75
	205,33	1,19	5,36	0,01	0,4998	94,33	1,51	6,71	54,06	2,50	9,50	8,00	101,23	2,97	0,33	13,36
	273,13	2,58	5,48	0,01	0,5005	114,18	0,96	6,71	58,20	3,57	9,50	8,03	127,23	2,42	0,86	23,96
	371,07	7,13	5,34	0,01	0,5001	173,58	1,06	6,74	53,22	4,44	9,50	8,03	173,00	3,40	0,75	16,78
	541,13	16,57	5,51	0,01	0,5007	268,07	7,76	6,73	50,46	6,13	9,50	8,03	229,17	11,86	1,24	20,27
	1012,53	66,84	5,82	0,01	0,4995	562,60	15,75	6,76	44,44	10,13	9,50	8,02	393,07	19,06	1,72	17,01
	1059,47	142,31	5,89	0,01	0,5009	585,60	36,06	6,77	44,73	10,64	9,50	8,07	417,43	40,44	1,74	16,31
	1513,50	216,57	6,22	0,01	0,5010	944,27	39,25	6,78	37,61	12,78	9,50	8,04	523,97	9,84	1,60	12,55
	2577,33	211,88	6,71	0,01	0,5002	1766,73	217,02	6,78	31,45	18,23	9,50	8,02	774,37	47,26	1,69	9,25
	2511,33	478,20	6,72	0,01	0,5000	1690,27	378,90	6,73	32,69	18,47	9,50	8,03	782,03	118,95	1,76	9,52
	5116,67	1409,02	7,47	0,01	0,4995	3776,67	881,94	6,78	26,19	30,18	9,50	8,08	1297,00	101,30	2,43	8,05
mean			6,13	0,01	0,5002			6,75	42,11	12,73	9,50	8,04			1,53	14,86
SD			0,71	0,00	0,0006			0,03	10,84	8,48	0,00	0,02			0,51	5,41

pure selenate adsorption

	c(Se)_init [µg/L]	SD [µg/L]	pH_init [-]	sol-Vol [mL]	m_kaolinite [g]	c(Se)_end [µg/L]	SD [µg/L]	pH_end [-]	c(Se)_loss [%]	c(Se)_sorp [µg/g]	Vol_sol [mL]	pH_desorp [-]	c(Se)_desorp [µg/L]	SD [µg/L]	c(Se)_fixed [µg/g]	desorp/sorp [%]
	12,77	0,53	5,79	0,01	0,4956	3,32	0,08	7,12	74,03	0,21	9,50	8,07	9,29	1,01	0,01	6,64
	32,38	0,77	5,80	0,01	0,4974	9,02	0,64	7,12	72,14	0,53	9,50	8,08	22,78	1,54	0,04	7,35
	61,04	1,33	5,85	0,01	0,4950	16,93	0,71	7,12	72,26	1,00	9,50	8,08	41,68	2,62	0,10	10,24
	90,87	1,65	5,84	0,01	0,4989	23,93	1,17	7,12	73,67	1,51	9,50	8,08	62,76	1,73	0,17	10,94
	109,38	2,32	5,84	0,01	0,4962	31,68	1,35	7,11	71,04	1,76	9,50	8,08	73,45	2,45	0,18	10,19
	182,23	1,83	5,80	0,01	0,4999	52,47	0,84	7,11	71,21	2,92	9,50	8,08	117,60	4,76	0,41	13,91
	243,47	2,21	5,81	0,01	0,4948	71,04	1,10	7,12	70,82	3,92	9,50	8,08	156,30	3,82	0,54	13,89
	306,73	3,85	5,79	0,01	0,5001	88,65	1,40	7,11	71,10	4,91	9,50	8,08	199,97	3,24	0,63	12,89
	428,73	4,07	5,76	0,01	0,5006	131,05	1,49	7,09	69,43	6,69	9,50	8,08	271,30	9,28	0,90	13,42
	617,67	6,57	5,72	0,01	0,4970	193,37	2,72	7,09	68,69	9,60	9,50	8,08	382,00	11,00	1,39	14,47
	933,40	2,97	5,73	0,01	0,4980	314,07	3,68	7,11	66,35	13,99	9,50	8,09	551,73	7,97	2,15	15,37
	1221,20	6,16	5,71	0,01	0,4980	424,53	2,80	7,09	65,24	17,99	9,50	8,09	703,50	13,28	2,90	16,11
	1704,33	9,54	5,78	0,01	0,4963	718,07	5,08	7,09	57,87	22,35	9,50	8,09	919,43	12,51	2,56	11,44
	2272,17	15,31	5,74	0,01	0,4977	989,13	7,04	7,06	56,47	29,00	9,50	8,09	1213,00	14,43	2,95	10,19
	2857,17	17,57	5,68	0,01	0,4977	1335,40	7,64	7,08	53,26	34,39	9,50	8,09	1470,00	12,38	2,83	8,23
	3954,33	36,58	5,77	0,01	0,5007	2094,67	9,82	7,12	47,03	41,78	9,50	8,09	1804,00	10,85	3,28	7,84
	5615,00	26,88	5,73	0,01	0,5002	3322,67	15,78	7,12	40,83	51,55	9,50	8,09	2253,67	16,93	3,40	6,60
mean			5,74	0,01	0,4985			7,09	58,35	25,26	9,50	8,09			2,48	11,52
SD			0,03	0,00	0,0016			0,02	10,04	15,16	0,00	0,00			0,85	3,51
