# Supplementary material for: Coupling Langmuir with Michaelis-Menten—A practical alternative to estimate Se content in rice?
Source: PLoS One. 2019 Apr 19;14(4):e0214219. doi: 10.1371/journal.pone.0214219 (PMC6474650; doi:10.1371/journal.pone.0214219)
Supplement: S2 Table — (PDF) [file pone.0214219.s002.pdf]

S2 Table: Experimental data of selenite sorption onto kaolinite in the presence of 0.1 M KCl and 750 µM nitrate, phosphate or sulfate

nitrate competition to selenite adsorption

	c(Se)_init [µg/L]	SD [µg/L]	c(N)_init [µg/L]	SD [µg/L]	pH_init [-]	sol-Vol [mL]	m_kaolinite [g]	c(Se)_end [µg/L]	SD [µg/L]	c(N)_end [µg/L]	SD [µg/L]	pH_end [-]	c(Se)_loss [%]	c(Se)_sorp [µg/g]	c(N)_loss [%]	c(N)_sorp [µg/g]
	11,49	0,01	10490,62	1,38	5,53	0,01	0,4987	3,48	0,00	10394,31	1,31	6,41	69,73	0,18	0,92	2,17
	30,17	0,11	10457,86	1,33	5,36	0,01	0,5008	9,48	0,01	10399,13	1,33	6,44	68,57	0,46	0,56	1,32
	58,24	0,21	10587,76	1,36	5,14	0,01	0,4999	19,76	0,08	10532,66	1,34	6,43	66,07	0,87	0,52	1,24
	117,89	1,55	10598,78	1,39	5,30	0,01	0,4995	42,65	0,36	10512,30	1,34	6,41	63,83	1,69	0,82	1,95
	295,40	8,52	10428,27	1,37	5,59	0,01	0,5007	120,15	1,69	10394,96	1,31	6,44	59,33	3,94	0,32	0,75
	552,93	27,14	10577,54	1,35	5,71	0,01	0,4992	246,87	3,21	10504,48	1,34	6,48	55,35	6,90	0,69	1,65
	1448,47	9,31	10495,66	1,36	6,15	0,01	0,5003	815,33	47,19	10441,01	1,32	6,42	43,71	14,24	0,52	1,23
	2676,00	486,86	10397,14	1,33	6,76	0,01	0,5005	1692,83	157,52	10378,01	1,33	6,44	36,74	22,10	0,18	0,43
	5206,67	541,93	10376,42	1,30	8,13	0,01	0,4999	3771,67	324,63	10304,75	1,30	6,57	27,56	32,29	0,69	1,61
mean	1155,25	119,51	10490,01	1,35	5,96	0,01	0,4999	746,91	59,41	10429,07	1,33	6,45	54,54	9,18	0,58	1,37
SD	1759,56	224,46	83,25	0,03	0,95	0,00	0,0007	1265,54	112,18	74,85	0,01	0,05	15,13	11,41	0,23	0,55

phosphate competition to selenite adsorption

	c(Se)_init [µg/L]	SD [µg/L]	c(P)_init [µg/L]	SD [µg/L]	pH_init [-]	sol-Vol [mL]	m_kaolinite [g]	c(Se)_end [µg/L]	SD [µg/L]	c(P)_end [µg/L]	SD [µg/L]	pH_end [-]	c(Se)_loss [%]	c(Se)_sorp [µg/g]	c(P)_loss [%]	c(P)_sorp [µg/g]
	12,09	0,06	24824,22	1328,58	5,54	0,01	0,4996	9,57	0,01	15311,08	592,42	6,43	20,84	0,06	38,32	214,18
	28,56	0,22	24688,47	1314,09	5,45	0,01	0,5010	24,67	0,05	15696,03	612,40	6,41	13,61	0,09	36,42	201,90
	59,39	0,55	24303,93	1273,48	5,35	0,01	0,4996	50,94	0,21	16259,63	640,10	6,41	14,23	0,19	33,10	181,12
	115,09	0,75	24344,06	1277,69	5,37	0,01	0,4998	103,59	0,94	16138,40	633,49	6,43	9,99	0,26	33,71	184,69
	287,13	5,34	24818,15	1327,94	5,47	0,01	0,4999	265,40	2,81	16182,75	642,37	6,39	7,57	0,49	34,79	194,30
	544,00	14,14	25078,39	1355,93	5,66	0,01	0,5004	505,53	1,54	17335,21	716,15	6,31	7,07	0,86	30,88	174,07
	1420,47	69,84	25008,90	1348,43	6,26	0,01	0,4996	1335,40	109,60	17623,43	733,58	6,47	5,99	1,92	29,53	166,28
	2631,83	255,28	25109,39	1359,28	6,84	0,01	0,5013	2520,33	405,09	18575,35	797,93	6,53	4,24	2,50	26,02	146,62
	5283,33	664,80	25022,26	1574,28	7,82	0,01	0,5006	5041,67	727,70	18757,78	836,86	6,49	4,57	5,43	25,04	140,78
mean	1153,54	112,33	24799,75	1351,08	5,97	0,01	0,5002	1095,23	138,66	16875,52	689,48	6,43	9,79	1,31	31,98	178,22
SD	1777,57	223,24	302,82	89,26	0,85	0,00	0,0006	1699,19	258,03	1247,21	86,09	0,06	5,49	1,77	4,51	24,30

sulphate competition to selenite adsorption

	c(Se)_init [µg/L]	SD [µg/L]	c(S)_init [µg/L]	SD [µg/L]	pH_init [-]	sol-Vol [mL]	m_kaolinite [g]	c(Se)_end [µg/L]	SD [µg/L]	c(S)_end [µg/L]	SD [µg/L]	pH_end [-]	c(Se)_loss [%]	c(Se)_sorp [µg/g]	c(S)_loss [%]	c(S)_sorp [µg/g]
	12,44	0,03	30658,83	1812,91	8,03	0,01	0,5000	6,12	0,01	29989,76	1735,47	7,63	50,76	0,14	2,18	15,05
	28,62	0,26	30569,18	1802,32	8,00	0,01	0,4996	11,34	0,01	29436,51	1673,53	7,65	60,37	0,39	3,71	25,50
	58,07	0,28	30662,62	1813,36	7,94	0,01	0,5003	23,45	0,05	29680,28	1700,78	7,57	59,61	0,78	3,20	22,09
	113,46	0,50	30722,55	1820,46	7,90	0,01	0,4994	46,50	0,22	29703,06	1703,52	7,49	59,02	1,51	3,32	22,96
	286,53	4,22	30683,98	1815,89	7,81	0,01	0,4996	131,80	1,78	29590,20	1690,88	7,44	54,00	3,48	3,56	24,63
	542,00	18,81	30653,40	1812,27	7,78	0,01	0,5007	229,40	0,46	29975,84	1733,89	7,44	57,68	7,02	2,21	15,22
	1676,17	100,69	30609,82	819,25	7,76	0,01	0,5008	1158,73	65,73	29909,73	765,42	7,45	30,87	11,62	2,29	15,73
	2639,50	507,20	30513,15	1795,72	7,83	0,01	0,4994	1817,67	269,65	30030,56	1739,81	7,43	31,14	18,51	1,58	10,87
	5250,00	694,51	30530,77	1797,80	7,98	0,01	0,5007	4083,00	409,83	28820,38	1656,64	7,51	22,23	26,22	5,60	38,43
mean	1178,53	147,39	30622,70	1698,89	7,89	0,01	0,5000	834,22	83,08	29681,81	1599,99	7,51	47,30	7,74	3,07	21,16
SD	1775,94	263,30	71,63	329,97	0,10	0,00	0,0006	1375,09	151,19	381,76	314,27	0,08	14,93	9,29	1,20	8,22
