# Supplementary material for: Coupling Langmuir with Michaelis-Menten—A practical alternative to estimate Se content in rice?
Source: PLoS One. 2019 Apr 19;14(4):e0214219. doi: 10.1371/journal.pone.0214219 (PMC6474650; doi:10.1371/journal.pone.0214219)
Supplement: S3 Table — (PDF) [file pone.0214219.s003.pdf]

S3 Table: Experimental data of selenate sorption onto kaolinite in the presence of 0.1 M KCl and 750 µM nitrate, phosphate or sulfate

nitrate competition to selenate adsorption

	c(Se)_init [µg/L]	SD [µg/L]	c(N)_init [µg/L]	SD [µg/L]	pH_init [-]	sol-Vol [mL]	m_kaolinite [g]	c(Se)_end [µg/L]	SD [µg/L]	c(N)_end [µg/L]	SD [µg/L]	pH_end [-]	c(Se)_loss [%]	c(Se)_sorp [µg/g]	c(N)_loss [%]	c(N)_sorp [µg/g]
	13,07	0,05	10166,23	41,61	5,52	0,01	0,5003	6,45	0,00	10138,55	42,43	6,81	50,64	0,15	0,27	0,62
	30,73	0,22	10433,86	43,70	5,40	0,01	0,5000	15,10	0,02	10373,28	44,53	6,81	50,87	0,35	0,58	1,36
	63,07	0,48	10145,03	41,41	5,25	0,01	0,5002	29,59	0,26	10109,34	42,22	6,78	53,09	0,75	0,35	0,80
	123,46	1,20	10485,41	43,96	5,41	0,01	0,5008	61,50	0,19	10383,91	43,96	6,76	50,19	1,39	0,97	2,28
	306,20	11,47	10195,54	41,91	5,40	0,01	0,4998	152,09	2,29	10181,16	43,14	6,77	50,33	3,47	0,14	0,32
	584,20	4,05	10316,35	42,78	5,43	0,01	0,5011	303,33	4,87	10270,53	43,19	6,75	48,08	6,30	0,44	1,03
	1478,53	96,47	10381,15	43,32	5,48	0,01	0,4997	834,47	34,38	10335,06	43,32	6,73	43,56	14,50	0,44	1,04
	2733,17	110,46	10265,95	42,22	5,43	0,01	0,4997	1695,50	77,70	10186,57	42,22	6,72	37,97	23,36	0,77	1,79
	5488,33	1267,48	10066,85	40,78	5,42	0,01	0,4995	3856,17	507,32	10032,92	41,99	6,71	29,74	36,75	0,34	0,76
mean	1202,31	165,76	10272,93	42,41	5,42	0,01	0,5001	772,69	69,67	10223,48	43,00	6,76	46,05	9,67	0,48	1,11
SD	1844,71	415,46	141,82	1,10	0,07	0,00	0,0005	1283,93	166,18	123,77	0,86	0,04	7,67	12,84	0,26	0,61

phosphate competition to selenate adsorption

	c(Se)_init [µg/L]	SD [µg/L]	c(P)_init [µg/L]	SD [µg/L]	pH_init [-]	sol-Vol [mL]	m_kaolinite [g]	c(Se)_end [µg/L]	SD [µg/L]	c(P)_end [µg/L]	SD [µg/L]	pH_end [-]	c(Se)_loss [%]	c(Se)_sorp [µg/g]	c(P)_loss [%]	c(P)_sorp [µg/g]
	12,85	0,02	24915,17	48,47	5,48	0,01	0,4999	12,62	0,03	18756,55	27,47	6,54	1,77	0,01	24,72	138,58
	30,82	0,07	25072,06	49,08	5,27	0,01	0,5007	29,29	0,11	19033,07	28,28	6,56	4,95	0,03	24,09	135,66
	61,91	0,11	25045,90	48,98	5,18	0,01	0,5001	58,90	0,41	19095,10	28,47	6,67	4,86	0,07	23,76	133,86
	120,77	0,87	25041,11	48,96	5,15	0,01	0,5008	116,39	0,74	18737,87	27,41	6,64	3,63	0,10	25,17	141,58
	305,00	2,80	24490,19	46,83	5,23	0,01	0,5007	292,53	3,81	18586,49	26,97	6,59	4,09	0,28	24,11	132,63
	631,73	18,32	24952,89	48,61	5,29	0,01	0,5002	605,13	12,24	19161,49	28,67	6,57	4,21	0,60	23,21	130,24
	1621,00	51,47	24466,62	46,74	5,31	0,01	0,5002	1577,93	109,75	18989,58	28,15	6,63	2,66	0,97	22,39	123,17
	3002,83	129,45	24520,61	46,94	5,26	0,01	0,4998	2944,83	273,22	18949,45	26,00	6,56	1,93	1,31	22,72	125,39
	6001,67	173,25	24728,15	47,74	5,25	0,01	0,5001	5811,67	1491,16	19225,93	28,86	6,61	3,17	4,27	22,25	123,75
mean	1309,84	41,82	24803,63	48,04	5,27	0,01	0,5003	1272,14	210,16	18948,39	27,81	6,60	3,47	0,85	23,60	131,65
SD	2022,84	65,22	254,82	0,99	0,09	0,00	0,0004	1963,48	488,99	213,58	0,93	0,04	1,18	1,36	1,03	6,56

sulphate competition to selenate adsorption

	c(Se)_init [µg/L]	SD [µg/L]	c(S)_init [µg/L]	SD [µg/L]	pH_init [-]	sol-Vol [mL]	m_kaolinite [g]	c(Se)_end [µg/L]	SD [µg/L]	c(S)_end [µg/L]	RSD [%]	pH_end [-]	c(Se)_loss [%]	c(Se)_sorp [µg/g]	c(S)_loss [%]	c(S)_sorp [µg/g]
	11,79	0,01	29604,62	21,03	8,46	0,01	0,4997	11,54	0,02	27823,66	16,32	7,47	2,07	0,01	6,02	40,09
	29,66	0,12	29813,67	21,32	8,42	0,01	0,5002	29,16	0,20	28103,68	16,80	7,47	1,69	0,01	5,74	38,46
	58,37	0,25	29201,71	20,46	8,33	0,01	0,4992	57,37	0,21	27904,67	16,48	7,43	1,72	0,02	4,44	29,23
	119,15	1,82	29580,30	20,99	8,28	0,01	0,4992	113,15	1,63	28163,88	16,88	7,43	5,04	0,14	4,79	31,92
	296,00	0,59	29576,92	20,99	8,24	0,01	0,5007	282,93	1,99	28290,41	17,36	7,45	4,41	0,29	4,35	28,90
	572,80	8,02	29154,21	20,39	8,20	0,01	0,5002	543,87	16,51	28093,74	16,85	7,42	5,05	0,65	3,64	23,85
	1476,27	154,16	29491,16	20,87	8,14	0,01	0,5000	1398,27	68,18	27970,25	16,62	7,42	5,28	1,75	5,16	34,22
	2683,67	109,29	29410,91	20,75	8,21	0,01	0,4997	2626,17	427,78	27370,66	15,39	7,44	2,14	1,29	6,94	45,93
	5405,00	1891,75	29278,84	20,57	8,22	0,01	0,5004	5258,33	995,39	27751,27	15,98	7,40	2,71	3,30	5,22	34,34
mean	1183,63	240,67	29456,93	20,82	8,28	0,01	0,4999	1146,75	167,99	27941,36	16,52	7,44	3,35	0,83	5,14	34,10
SD	1817,31	621,81	215,20	0,30	0,11	0,00	0,0005	1769,87	339,99	273,58	0,58	0,02	1,56	1,12	0,99	6,67
