# Supplementary material for: Coupling Langmuir with Michaelis-Menten—A practical alternative to estimate Se content in rice?
Source: PLoS One. 2019 Apr 19;14(4):e0214219. doi: 10.1371/journal.pone.0214219 (PMC6474650; doi:10.1371/journal.pone.0214219)
Supplement: S4 Table — (PDF) [file pone.0214219.s004.pdf]

S4 Table: Experimental data of selenite and selenate sorption onto kaolinite in the presence of nutrient solution

nutrient solution competition to selenite adsorption

	c(Se)_init [µg/L]	SD [µg/L]	pH_init [-]	sol-Vol [mL]	m_kaolinite [g]	c(Se)_end [µg/L]	SD [µg/L]	pH_end [-]	c(Se)_loss [%]	c(Se)_sorp [µg/g]
	23,01	2,93	4,92	0,17	8,0263	18,31	6,10	6,59	20,41	0,11
	23,19	0,80	4,94	0,17	8,4421	18,24	0,26	6,68	21,34	0,11
	55,24	6,72	5,06	0,17	8,0056	34,08	10,37	6,51	38,31	0,51
	59,63	2,36	5,08	0,17	8,4133	46,37	0,96	6,64	22,24	0,30
	105,83	7,39	5,14	0,17	8,0032	83,70	12,35	6,53	20,91	0,53
	120,15	3,99	5,23	0,17	8,6784	96,23	2,76	6,62	19,90	0,53
	211,80	13,13	5,12	0,17	8,0166	189,37	24,63	6,52	10,59	0,54
	233,00	6,76	5,17	0,17	8,4258	193,75	5,20	6,66	16,85	0,89
	521,40	23,30	5,13	0,17	8,0054	414,35	30,11	6,52	20,53	2,56
	555,53	15,90	5,19	0,17	8,4503	476,73	15,63	6,63	14,18	1,78
	1068,53	55,99	5,30	0,17	8,0009	912,00	89,65	6,51	14,65	3,74
	1182,73	35,99	5,16	0,17	8,4962	1019,27	29,00	6,65	13,82	3,68
	2110,67	86,00	5,54	0,17	8,0122	1841,05	114,77	6,57	12,77	6,43
	2376,67	73,66	5,42	0,17	8,5001	2044,20	59,08	6,74	13,99	7,48
	5370,00	155,86	5,83	0,17	8,6087	4778,63	152,61	6,59	11,01	13,14
	5706,67	170,39	5,96	0,17	8,4301	5178,33	134,29	6,76	9,26	11,98
mean	3335,13	103,44	5,48	0,17	8,1243	3031,27	101,69	6,62	15,77	7,04
SD	4209,06	117,48	0,47	0,00	0,2795	3891,55	101,46	0,13	9,04	7,46

nutrient solution competition to selenite adsorption

	c(Se)_init [µg/L]	SD [µg/L]	pH_init [-]	sol-Vol [mL]	m_kaolinite [g]	c(Se)_end [µg/L]	SD [µg/L]	pH_end [-]	c(Se)_loss [%]	c(Se)_sorp [µg/g]
	20,59	1,73	4,92	0,17	8,0263	19,51	0,71	6,59	5,26	0,03
	27,47	0,88	4,80	0,17	8,5647	24,13	0,98	6,71	12,16	0,07
	54,47	4,39	5,06	0,17	8,0056	51,33	1,73	6,51	5,77	0,08
	66,38	2,32	5,04	0,17	8,5480	61,41	1,99	6,78	7,48	0,11
	110,00	8,00	5,14	0,17	8,0032	104,32	3,22	6,53	5,16	0,14
	123,57	3,40	5,14	0,17	8,5118	104,25	3,56	6,74	15,63	0,43
	213,70	15,41	5,12	0,17	8,0166	195,98	6,12	6,52	8,29	0,42
	265,47	7,60	4,88	0,17	8,5470	241,40	8,16	6,71	9,07	0,54
	534,60	41,89	5,13	0,17	8,0054	485,18	16,69	6,52	9,25	1,18
	621,93	20,51	4,86	0,17	8,4568	538,47	19,30	6,64	13,42	1,89
	1028,15	81,20	5,30	0,17	8,0009	961,65	33,09	6,51	6,47	1,59
	1254,60	42,12	5,04	0,17	8,5207	1102,93	38,88	6,68	12,09	3,40
	2062,00	153,94	5,54	0,17	8,0122	1844,75	59,33	6,57	10,54	5,19
	2458,00	81,46	5,07	0,17	8,3806	2203,33	68,71	7,04	10,36	5,81
	5161,25	385,92	5,83	0,17	8,0087	4739,19	153,59	6,59	8,18	10,08
	6285,00	199,44	5,14	0,17	8,5390	5790,00	191,57	6,70	7,88	11,09
mean	3493,61	183,37	5,28	0,17	8,1017	3210,27	103,47	6,58	7,53	6,58
SD	4504,69	236,56	0,35	0,00	0,2061	4144,91	132,58	0,07	1,69	8,29
