# Supplementary material for: Coupling Langmuir with Michaelis-Menten—A practical alternative to estimate Se content in rice?
Source: PLoS One. 2019 Apr 19;14(4):e0214219. doi: 10.1371/journal.pone.0214219 (PMC6474650; doi:10.1371/journal.pone.0214219)
Supplement: S5 Table — (PDF) [file pone.0214219.s005.pdf]

S5 Table: Experimental data of selenite uptake into rice seedlings in the presence of nutrient solution and kaolinite

c(Se) added selenite [µg/L] solution-Se	c(Se) selenite in solution [µg/L] solution-Se	dry weight shoot [g] shoot weight	SD dry weight shoot [g] shoot weight	dry weight root [g] root weight	SD dry weight root [g] root weight	c(Se) in shoot tissue [mg/kg] shoot-Se	SD shoot tissue [mg/kg] SD shoot	c(Se) in root tissue [mg/kg] root-Se	SD root tissue [mg/kg] SD root	c(Se) in plant tissue [mg/kg] plant-Se	SD planttissue [mg/kg] SD plant
20	23	0.01320	0.00176	0.00618	0.00095	1.56	0.11	3.38	0.47	2.14	0.23
50	60	0.01330	0.00075	0.00614	0.00062	4.19	0.19	9.25	0.29	5.79	0.24
100	120	0.01257	0.00141	0.00618	0.00031	9.28	0.70	15.60	4.03	11.36	1.29
200	233	0.01173	0.00121	0.00668	0.00069	19.16	0.73	36.03	1.06	25.28	0.85
500	556	0.01473	0.00145	0.00683	0.00025	39.57	0.95	110.00	5.68	61.87	1.64
1000	1183	0.01145	0.00071	0.00695	0.00034	71.18	4.04	166.71	5.31	107.26	4.45
2000	2377	0.01082	0.00097	0.00780	0.00053	129.00	6.44	264.68	25.20	185.84	13.09
5000	5707	0.00960	0.00184	0.00621	0.00079	93.72	3.79	323.45	9.77	183.99	5.59
10000	11378	0.01102	0.00257	0.00978	0.00512	87.93	6.12	487.98	18.05	276.03	14.06
20	23	0.00863	0.00205	0.00542	0.00122	4.24	0.44	14.54	0.46	8.21	0.44
50	55	0.00765	0.00254	0.00464	0.00112	7.61	0.96	13.58	0.40	9.86	0.79
100	106	0.00948	0.00182	0.00553	0.00106	7.15	0.48	17.49	0.41	10.97	0.45
200	212	0.00667	0.00343	0.00633	0.00197	17.25	0.62	25.62	0.42	21.33	0.55
500	521	0.00936	0.00176	0.00511	0.00082	33.15	0.88	64.22	0.75	44.12	0.83
1000	1069	0.00558	0.00252	0.00481	0.00163	58.72	1.10	113.23	1.18	83.96	1.13
2000	2111	0.00809	0.00220	0.00530	0.00130	97.78	0.81	187.37	1.63	133.25	1.12
5000	5370	0.00399	0.00244	0.00438	0.00103	96.20	1.09	279.70	1.61	192.20	1.24
10000	10135	0.00466	0.00293	0.00476	0.00256	118.77	1.57	373.36	1.75	247.42	1.66
5	6	0.01070	0.00280	0.00572	0.00074	0.39	0.07	1.46	0.05	0.76	0.06
10	12	0.00920	0.00148	0.00530	0.00089	1.07	0.06	2.04	0.23	1.42	0.13
25	29	0.00803	0.00195	0.00570	0.00179	1.71	0.10	3.34	0.10	2.39	0.10
50	58	0.01137	0.00426	0.00527	0.00062	3.92	0.70	8.93	1.26	5.51	0.77
100	114	0.01059	0.00143	0.00504	0.00071	11.56	1.19	22.70	2.27	15.15	1.55
250	284	0.01083	0.00246	0.00500	0.00052	24.03	1.86	52.17	1.94	32.92	1.87
500	562	0.00880	0.00240	0.00528	0.00109	69.31	1.89	180.94	2.93	111.19	2.22
1000	1152	0.00918	0.00123	0.00622	0.00083	134.55	0.65	208.96	3.89	164.59	1.96
2500	2862	0.00870	0.00130	0.00527	0.00075	139.55	3.15	296.76	1.90	198.87	2.69
5	8	0.00763	0.00175	0.00366	0.00074	0.25	0.08	0.45	0.20	0.31	0.11
10	14	0.00703	0.00192	0.00450	0.00116	0.50	0.11	0.96	0.11	0.68	0.11
25	32	0.00920	0.00174	0.00414	0.00066	2.51	0.14	8.47	0.27	4.36	0.18
50	59	0.00905	0.00117	0.00445	0.00033	4.40	0.18	36.05	2.19	14.83	0.62
100	122	0.00789	0.00162	0.00429	0.00101	8.08	0.24	43.47	1.07	20.55	0.56
250	312	0.00888	0.00180	0.00416	0.00047	28.96	1.22	62.50	1.61	39.67	1.30
500	627	0.00792	0.00136	0.00468	0.00066	52.00	3.17	122.98	2.79	78.35	3.05
1000	1193	0.00774	0.00140	0.00447	0.00078	91.45	2.44	189.52	3.60	127.32	2.85
2500	3014	0.00756	0.00143	0.00514	0.00054	143.41	4.57	313.40	10.11	212.18	6.09
5	6	0.00922	0.00535	0.00660	0.00331	0.60	0.05	1.30	0.28	0.89	0.13
10	14	0.01193	0.00578	0.00905	0.00427	1.34	0.10	2.80	0.16	1.97	0.13
25	33	0.00908	0.01008	0.01193	0.00326	2.84	0.17	4.73	0.49	3.91	0.25
50	63	0.01070	0.00744	0.00942	0.00584	8.41	0.27	21.07	0.28	14.33	0.27
100	129	0.01130	0.01146	0.01130	0.00170	9.10	0.33	13.94	0.35	11.52	0.34
250	290	0.00868	0.00554	0.01193	0.00646	32.27	1.25	53.70	0.89	44.68	1.05
500	620	0.01590	0.00141	0.00750	0.00042	56.70	0.96	116.48	3.90	75.86	1.64
1000	1209	0.01020	0.00577	0.01150	0.00719	79.71	3.89	135.95	4.73	109.51	4.35
2500	2934	0.01163	0.00571	0.00797	0.00012	193.99	4.48	325.98	8.15	247.64	4.55
