# Supplementary material for: Coupling Langmuir with Michaelis-Menten—A practical alternative to estimate Se content in rice?
Source: PLoS One. 2019 Apr 19;14(4):e0214219. doi: 10.1371/journal.pone.0214219 (PMC6474650; doi:10.1371/journal.pone.0214219)
Supplement: S6 Table — (PDF) [file pone.0214219.s006.pdf]

S6 Table: Experimental data of selenate uptake into rice seedlings in the presence of nutrient solution and kaolinite

c(Se) added selenate [µg/L] solution-Se	c(Se) selenate in solution [µg/L] solution-Se	dry weight shoot [g] shoot weight	SD dry weight shoot [g] shoot weight	dry weight root [g] root weight	SD dry weight root [g] root weight	c(Se) in shoot tissue [mg/kg] shoot-Se	SD shoot tissue [mg/kg] SD shoot	c(Se) in root tissue [mg/kg] root-Se	SD root tissue [mg/kg] SD root	c(Se) in plant tissue [mg/kg] plant-Se	SD plant tissue [mg/kg] SD plant
20	27	0.01223	0.00212	0.00581	0.00041	0.96	0.06	0.30	0.12	0.75	0.07
50	66	0.01249	0.00212	0.00623	0.00075	5.28	0.20	1.84	0.29	4.14	0.22
100	124	0.01243	0.00092	0.00595	0.00037	13.44	0.52	3.59	0.73	10.25	0.58
200	265	0.01242	0.00339	0.00638	0.00035	27.33	1.20	8.11	1.51	20.81	1.23
500	622	0.01226	0.00242	0.00677	0.00203	66.41	2.77	27.58	0.89	52.59	1.91
1000	1255	0.01277	0.00145	0.00558	0.00025	139.17	5.52	54.32	2.63	113.36	5.09
2000	2458	0.01314	0.00266	0.00610	0.00091	297.46	20.24	94.10	5.92	232.99	16.60
5000	6285	0.01119	0.00293	0.00619	0.00109	746.37	23.46	234.40	10.80	564.07	20.02
10000	12430	0.01086	0.00160	0.00470	0.00057	1187.60	25.93	441.75	47.31	962.27	31.55
20	20	0.00863	0.00250	0.00525	0.00120	2.36	0.19	0.51	0.02	1.66	0.14
50	54	0.00838	0.00381	0.00425	0.00217	3.89	0.25	0.60	0.07	2.78	0.19
100	109	0.00817	0.00308	0.00583	0.00167	10.81	0.37	1.66	0.10	7.00	0.28
200	213	0.00950	0.00260	0.00500	0.00100	44.59	1.32	4.98	0.62	30.93	1.12
500	535	0.00900	0.00255	0.00513	0.00105	104.46	1.81	10.12	1.73	70.23	1.79
1000	1035	0.00817	0.00372	0.00550	0.00206	183.16	4.11	23.92	1.17	119.08	3.06
2000	2056	0.00871	0.00286	0.00557	0.00090	375.27	8.86	66.31	2.95	254.77	7.44
5000	4210	0.00760	0.00301	0.00560	0.00049	650.65	17.77	92.84	4.48	414.00	15.91
10000	10635	0.00467	0.00176	0.00778	0.00413	1264.99	26.77	296.23	4.45	659.51	11.13
5	6	0.01139	0.00440	0.00504	0.00090	0.29	0.05	0.00	0.00	0.20	0.04
10	12	0.01207	0.00453	0.00496	0.00105	0.79	0.12	0.49	0.06	0.70	0.11
25	28	0.01504	0.00261	0.00504	0.00061	1.93	0.14	1.17	0.17	1.74	0.14
50	57	0.01476	0.00538	0.00432	0.00058	7.37	0.66	3.34	0.73	6.46	0.66
100	112	0.01238	0.00190	0.00662	0.00114	13.30	0.33	4.61	0.65	10.27	0.45
250	288	0.01190	0.00363	0.00507	0.00120	33.03	1.16	18.06	2.34	28.56	1.45
500	572	0.01306	0.00263	0.00454	0.00078	67.38	1.23	39.23	3.07	60.12	1.65
1000	1138	0.01168	0.00396	0.00690	0.00197	191.77	5.37	40.66	2.79	135.65	4.52
2500	2808	0.01366	0.00550	0.00486	0.00083	424.73	13.20	157.26	2.84	354.54	11.84
5	5	0.00776	0.00314	0.00738	0.00335	0.34	0.06	0.00	0.00	0.18	0.03
10	14	0.00772	0.00456	0.00712	0.00169	0.55	0.07	0.16	0.09	0.37	0.07
25	27	0.00945	0.00196	0.00599	0.00152	2.20	0.28	1.18	0.14	1.81	0.22
50	56	0.00968	0.00232	0.00603	0.00194	4.62	0.11	2.04	0.16	3.63	0.13
100	82	0.00670	0.00262	0.00643	0.00280	3.43	0.12	1.96	0.18	2.71	0.15
250	311	0.00752	0.00271	0.00567	0.00289	35.87	1.13	7.59	0.22	23.72	0.66
500	679	0.00902	0.00205	0.00611	0.00218	96.02	2.76	28.95	0.59	68.93	1.64
1000	1322	0.00778	0.00333	0.00810	0.00269	197.57	3.91	85.19	2.10	140.23	3.10
2500	3285	0.00816	0.00366	0.00823	0.00371	451.62	6.21	99.56	4.01	274.82	5.10
5	7	0.01207	0.00514	0.00796	0.00351	1.11	0.12	1.08	0.11	1.10	0.12
10	13	0.01220	0.00375	0.00790	0.00215	0.92	0.16	0.00	0.20	0.56	0.17
25	32	0.01395	0.00373	0.00903	0.00431	2.46	0.12	0.00	0.14	1.50	0.13
50	66	0.00946	0.00506	0.00454	0.00154	5.27	0.53	2.11	0.19	4.24	0.45
100	127	0.00930	0.00481	0.00605	0.00064	10.50	1.31	3.30	0.47	7.66	1.21
250	314	0.01377	0.00261	0.00757	0.00159	52.84	2.25	26.47	1.30	43.49	1.89
500	618	0.00963	0.00588	0.00960	0.00624	81.51	1.23	21.94	2.68	51.78	1.98
1000	1240	0.01208	0.00636	0.00693	0.00180	183.78	6.52	63.37	5.54	139.89	6.31
2500	3252	0.00500	0.00157	0.01283	0.00943	339.67	6.04	177.08	8.86	222.67	8.46
